# Liver Transplantation for Non-Resectable Liver Metastases from Colorectal Cancer: A Systematic Review and Meta-Analysis

**DOI:** 10.1007/s00268-021-06248-4

**Published:** 2021-07-28

**Authors:** Rebecca Varley, Munir Tarazi, Madhav Davé, Shahd Mobarak, Martyn C. Stott, Minas Baltatzis, Thomas Satyadas

**Affiliations:** 1 Department of Hepato-Pancreato-Biliary Surgery, Manchester Royal Infirmary, Manchester University NHS Foundation Trust, Oxford Road, Manchester, M13 9WL UK; 2 Department of Upper GI Surgery, Salford Royal Foundation Trust, Salford, UK

## Abstract

**Backgrounds:**

Colorectal liver metastases were historically considered a contraindication to liver transplantation, but dismal outcomes for those with metastatic colorectal cancer and advancements in liver transplantation (LT) have led to a renewed interest in the topic. We aim to compare the current evidence for liver transplantation for non-resectable colorectal liver metastases (NRCLM) with the current standard treatment of palliative chemotherapy.

**Methods:**

A systematic review and meta-analysis of proportions was conducted following screening of MEDLINE, EMBASE, SCOPUS and CENTRAL for studies reporting liver transplantation for colorectal liver metastases. Post-operative outcomes measured included one-, three- and five-year survival, overall survival, disease-free survival and complication rate.

**Results:**

Three non-randomised studies met the inclusion criteria, reporting a total of 48 patients receiving LT for NRCLM. Survival at one-, three- and five-years was 83.3–100%, 58.3–80% and 50–80%, respectively, with no significant difference detected (*p* = 0.22, *p* = 0.48, *p* = 0.26). Disease-free survival was 35–56% with the most common site of recurrence being lung. Thirteen out of fourteen deaths were due to disease recurrence.

**Conclusion:**

Although current evidence suggests a survival benefit conferred by LT in NRCLM compared to palliative chemotherapy, the ethical implications of organ availability and allocation demand rigorous justification. Concomitant improvements in the management of patients following liver resection and of palliative chemotherapy regimens is paramount.

## Introduction

Colorectal cancer (CRC) is the third most common cancer and is responsible for one in four cancer deaths worldwide [1]. 40–50% of patients will develop secondary liver metastases, the presence of which reduces overall survival by a factor of nine [2]. The incidence of CRC is increasing in younger patients who are more likely to present with or develop liver metastases [3].

Liver resection with neoadjuvant and adjuvant chemotherapy is the gold standard for CRLM, however up to 80% of cases are non-resectable at presentation. Non-resectable CRLM (NRCLM) treated with palliative chemotherapy has a five-year survival of less than 10% [4, 5]. Of the 20–40% who are eligible for a liver resection [2], the median 5-year survival after resection is 38% (16–74%) and 40–75% of these patients will develop recurrent disease, predominantly in the liver [6].

Colorectal liver metastases (CRLM) were historically considered a contraindication to liver transplantation (LT), with a reported five-year survival of 18% from 1977 to 1995 [7, 8]. Dismal outcomes for those with unresectable metastatic colorectal cancer have led to a renewed interest in the topic, particularly for patients with liver-limited metastases. The last decade has seen a rapid increase in the number of registered trials and the success of trials reported from Norway has generated great enthusiasm. Despite this, there remains little high-level evidence to support LT for CRLM.

The European Liver Transplant Registry (ELTR) has reported 80,347 liver transplants from 1988–2009 with LT for secondary liver tumours representing 0.5% of these—the vast majority being for neuroendocrine tumour (NET) metastases [9, 10]. Transplantation for primary and secondary liver malignancies is becoming increasingly common, accounting for 12% of all liver transplants prior to 1997 with a recent increase to 24% [10].

LT for CRLM was originally abandoned, as the poor initial results could not justify the allocation of a scarce supply of organs. In the intervening period, the demand for LT has increased with only a modest corresponding increase in the donor pool and a high waiting-list mortality worldwide [11].

The aim of this systematic review is to evaluate the available evidence for survival and outcomes in patients with NRCLM who have undergone liver transplantation, compared with palliative chemotherapy.

## Materials and methods

### Design

A systematic review and meta-analysis of proportions was conducted in accordance with the PRISMA standards, registered on PROSPERO (CRD42020212716) with methods established prior to conducting review.

### Data sources and search strategy

Eligible studies were identified from MEDLINE/PubMed, EMBASE, SCOPUS and CENTRAL (The Cochrane Library) with a combination of the following search terms: colorectal/colonic/rectal neoplasm, liver/hepatic metastasis/metastases and liver transplant/transplantation. Reference lists of identified studies were screened manually for relevant citations. In addition, the World Health Organization International Clinical Trials Registry, ClinicalTrials.gov, ISRCTN Register and PROSPERO were searched to identify ongoing and unpublished studies.

### Study selection

Studies reporting LT for CRLM were included in final analysis. The following strict exclusion criteria were applied: non-English language publication, those including children under 18 years of age, non-human studies, case reports and series containing fewer than five patients, conference abstracts, the use of extended criteria or non-standard donors, and studies reporting other indications for LT.

Two authors (MT, MD) independently reviewed all studies identified by the search strategy. After removing duplicates, the titles and abstracts of the studies were screened for inclusion using Rayyan software [12]. Where there was uncertainty from the study abstract, the full paper was assessed for relevance. Conflicts were resolved through discussion and involvement of a third author (MS) where necessary.

### Data extraction

Two authors (RV, SM) independently extracted data from the studies. Disagreements were resolved through discussion and where consensus could not be reached, a third independent author (MS) was consulted. Extracted variables included study characteristics, patient demographics, disease and treatment details, outcome measures and follow up. Outcome measures included overall survival, one, three, and five-year survival, post-operative morbidity, 30-day mortality, overall mortality, disease-free survival and disease recurrence.

### Assessment of risk of bias

The assessment of methodological quality and risk of bias was carried out by two independent authors (MT, RV). The Cochrane Risk Of Bias In Non-Randomized Studies–of Interventions (ROBINS-I) tool was utilized.

### Data synthesis and statistical analysis

Data synthesis was done using the software Review Manager (RevMan) [13]. Descriptive analysis was performed of study characteristics, baseline patient demographics, and intervention details. All cohort studies included were non-comparative single arm studies; thus, a meta-analysis of proportions was conducted for data to calculate pooled outcome measures. This statistical analysis was performed using MedCalc for Windows, version 19.0 and was carried out using a Freeman-Turkey transformation [14] to calculate weighted summary proportion under the fixed and random effects model [15]. Statistical heterogeneity was assessed using Cochran Q test (*χ*^*2*^) and was further quantified by generating an inconsistency statistic (*I*^*2*^) for each outcome measure with the threshold for heterogeneity considered present if the *P* value was < 0.05 or *I*^*2*^ was greater than 50%. Kaplan–Meier curves from all included studies were combined to give overall survival (OS) and disease-free survival (DFS) curves (SPSS software).

## Results

### Study selection

**Fig. 1:**
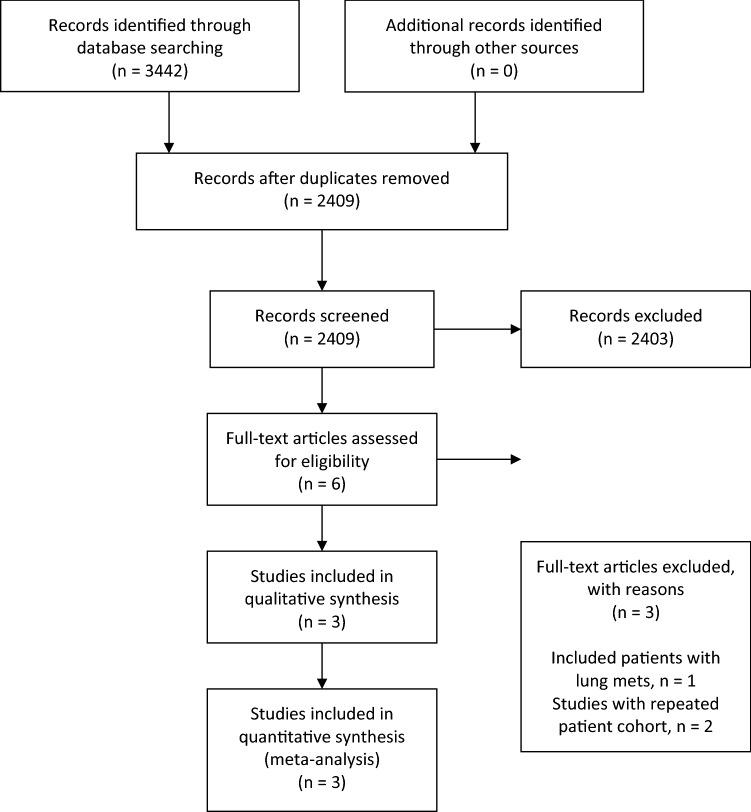
PRISMA flow diagram

The literature search identified 3442 studies. Duplicates were removed, and 2409 studies were assessed for eligibility. Following abstract screening, 2403 studies were excluded as irrelevant. From the remaining six, three studies met the inclusion criteria and comprise the study population for this systematic review: two prospective cohort studies and one retrospective cohort study reporting a total of 48 patients. (PRISMA flowchart, Fig. 1) [16–18].

### Methodological quality of included studies

**Fig. 2:**
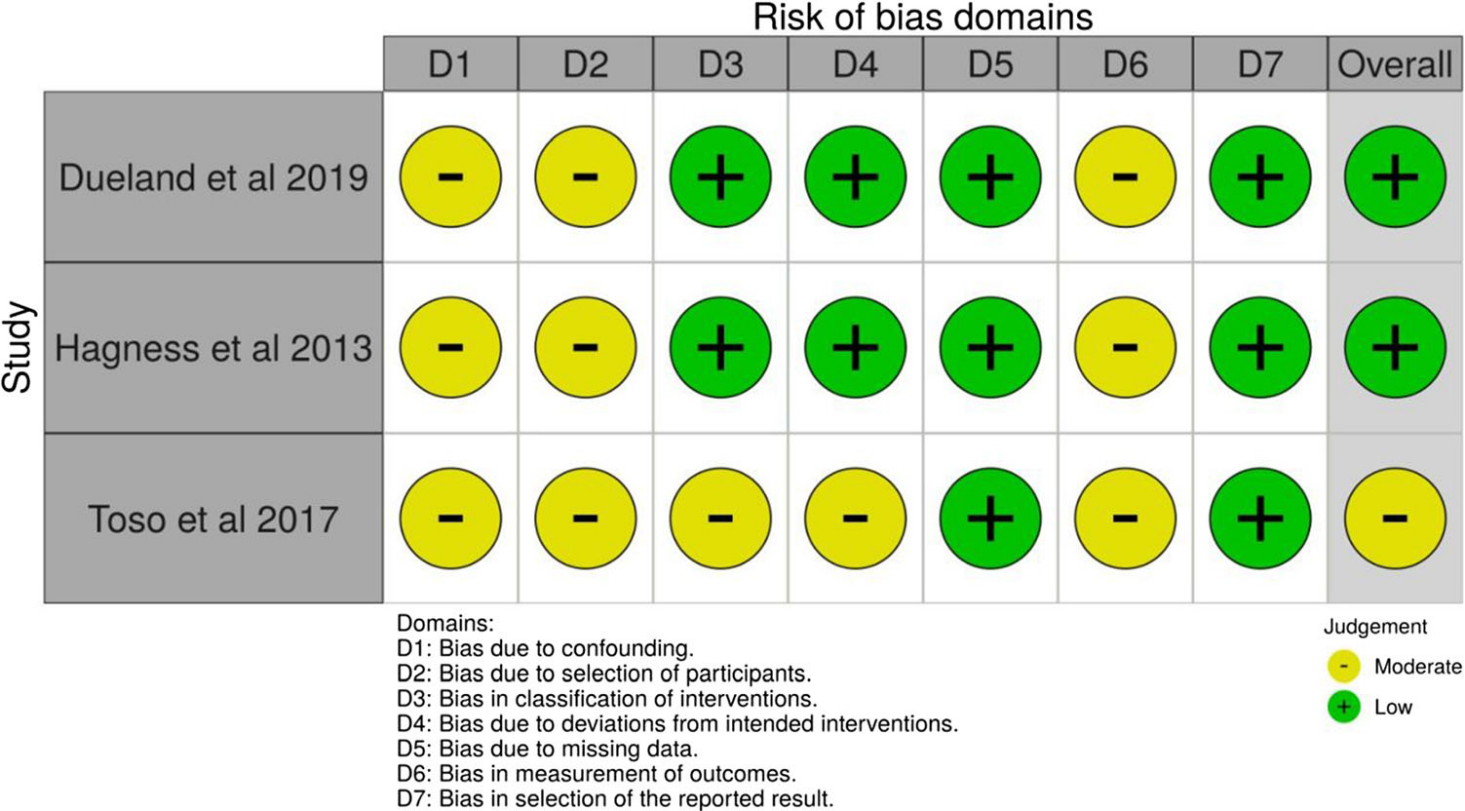
Risk of bias summary and graph showing authors’ judements about each risk of bias domain for observational studies using the ROBINS-I tool

The observational studies included varied in sample size, conduct, and reporting of outcomes (Fig. 2). Confounding bias was present in the included studies. The authors were able to measure and attempt to control for known confounders. Selection bias was present in all studies, as selection of participants into the study may have been related to the intervention and outcome. The included studies made reasonable efforts at reducing unintended deviation from interventions to limit attrition bias by performing appropriate analyses. Nevertheless, there remained considerable methodological heterogeneity.

### Study characteristics

The three eligible studies included a total of 48 patients transplanted between 1995 and 2016. One retrospective, multicentre study reported a series from centres in France, Portugal and Switzerland (*n* = 12). Two prospective single-arm studies reported cases from Olso, Norway (SECA-I (*n* = 21) and SECA-II trials (*n* = 15)). Study characteristics are outlined in Table 1. Sources of funding were not reported by any study. All results are reported in the order; Toso et al. [16], Hagness et al. (SECA-I) [17] Dueland et al. (SECA-II) where not otherwise specified [18].

**Table 1:** Summary of characteristics of included studies

Study characteristics	Toso et al. [16]]	Hagness (SECA-I) [17]]	Dueland (SECA-II) [18]]
	Retrospective	Prospective	Prospective
	Multicentre	Single centre	Single centre
	1995–2015	2006–2011	2012–2016
n	12	21	15
Male:female	6:6	13:8	8:7
Median age (range)	56 (38–73)	56 (45–46)	59.4 (34.9–71.1)
Performance status, ECOG 0–1	NR	21	15
Site of primary Ca: colon, rectum	11, 1	11, 10	11, 4
Node positive at primary diagnosis	7 (58.3%)	14 (33.3%)	7 (46.7%)
Liver metastases < 12 months from primary diagnosis	9 (75.0%)	17 (80.1%)	14 (93.3%)
Median lines of chemotherapy prior to LT (range)	2 (1–4)	2 (1–3)	2 (1–3)
Previous liver resection	10 (83.3%)	4 (19.0%)	4 (26.7%)
Previous ablation	1 (8.3%)	2 (9.5%)	2 (13.3%)
Median months from primary diagnosis to liver metasasis (range)	NR	36 (16–59)	24 (13.3–112.2)
Median months from primary resection to liver metastases (range)	41 (12–97)	NR	22.6 (2.3–111.2)
Median number of metastatic lesions at time of LT (range)	9 (1—> 15)	8 (4–40)	5 (1–53)
Median size of largest lesion at time of LT, mm (range)	150 (10–600) *	45 (28–130)	24 (3–47)
Median CEA at LT, unit (range)	16.9 (1–314)	15 (1–2002)	2 (1–30)
Median follow up, months (range)	26 (0–108)	27 (8–60)	26 (5–60)

*ECOG (Eastern cooperative oncology group), LT (liver transplantation), CEA (carcinoembryonic antigen)*

### Study participants

Tosa et al. [16] describe six patients undergoing planned transplantation; detailed selection criteria used were not reported. The remaining six in the study underwent “compassioned” transplantations following surgical complications (*n* = 3) or due to extensive disease burden (*n* = 3). No pre-operative investigation details were described. Eight patients received an mTOR inhibitor.

SECA-I [17] included patients with WHO performance status 0 or 1, completed radical excision of the primary tumour, minimum of six weeks chemotherapy and absence of extrahepatic disease. Patients were excluded if they had greater than 10% weight loss, standard contra-indications for LT, or had other malignancies. Pre-operative investigations included computed tomography (CT) of thorax, abdomen and pelvis (TAP), positron emission tomography (PET)/CT, bone scan, repeat CT TAP at time of LT, staging laparotomy if negative CT TAP and included frozen section of lymph nodes in the hepatoduodenal ligament. All patients received sirolimus, mycofenolate mofetil and corticosteroids.

SECA-II [18]: as for SECA-I plus no liver metastasis larger than 10 cm prior to chemotherapy with at least 10% response by RECIST criteria, if more than 30 lesions all less than five centimetres (cm) and at least 30% response by RECIST criteria, at least one-year time span from CRC diagnosis and being listed for transplant. Exclusion criteria as above and including BMI greater than 30. Pre-operative investigations included: PET/CT, CT or MR TAP within four weeks, colonoscopy or CT colonography within 12 months. All patients received tacrolimus converted to sirolimus after four to six weeks, mycofenolate mofetil and corticosteroids.

Liver transplantation technique was not described in detail by any study. Fifty per cent of patients reported by Toso et al. underwent living donor transplantation, while all patients in the SECA-I and SECA-II trials received deceased donor organs. No living or deceased donor details are available.

### Patient and primary tumour/metastasis characteristics

All patients had a primary diagnosis of colorectal adenocarcinoma with non-resectable liver metastases. The patient populations were heterogeneous both within and between studies. Baseline demographics, primary and metastatic disease details and treatment prior to LT are presented in Table 1. The median age was 56, 56 and 59 years. The colon was the most common site of primary tumour in all studies. The percentage of cases that were node positive at the time of diagnosis was 33.3—58.3% and 75.0%. From all studies, 93.3% had liver metastases confirmed within 12 months of primary diagnosis.

Of note, there was significant heterogeneity in patients who underwent liver resection prior to LT reported by Toso et al.–83.3% compared to 26.7 and 19.0% in the remaining two studies (*p* = 0.0006, *I*^*2*^ 86.5%). No significant difference was detected in patients receiving ablation prior to LT (*p* = 0.92). Four out of twelve (33.3%) patients received adjuvant chemotherapy following LT compared to none in the SECA-I or SECA-II trials (*p* = 0.006). At the time of LT, the median number of metastatic lesions was 9, 8 and 5 with a median size of 150, 45 and 24 mm.

### Major complications

**Fig. 3:**
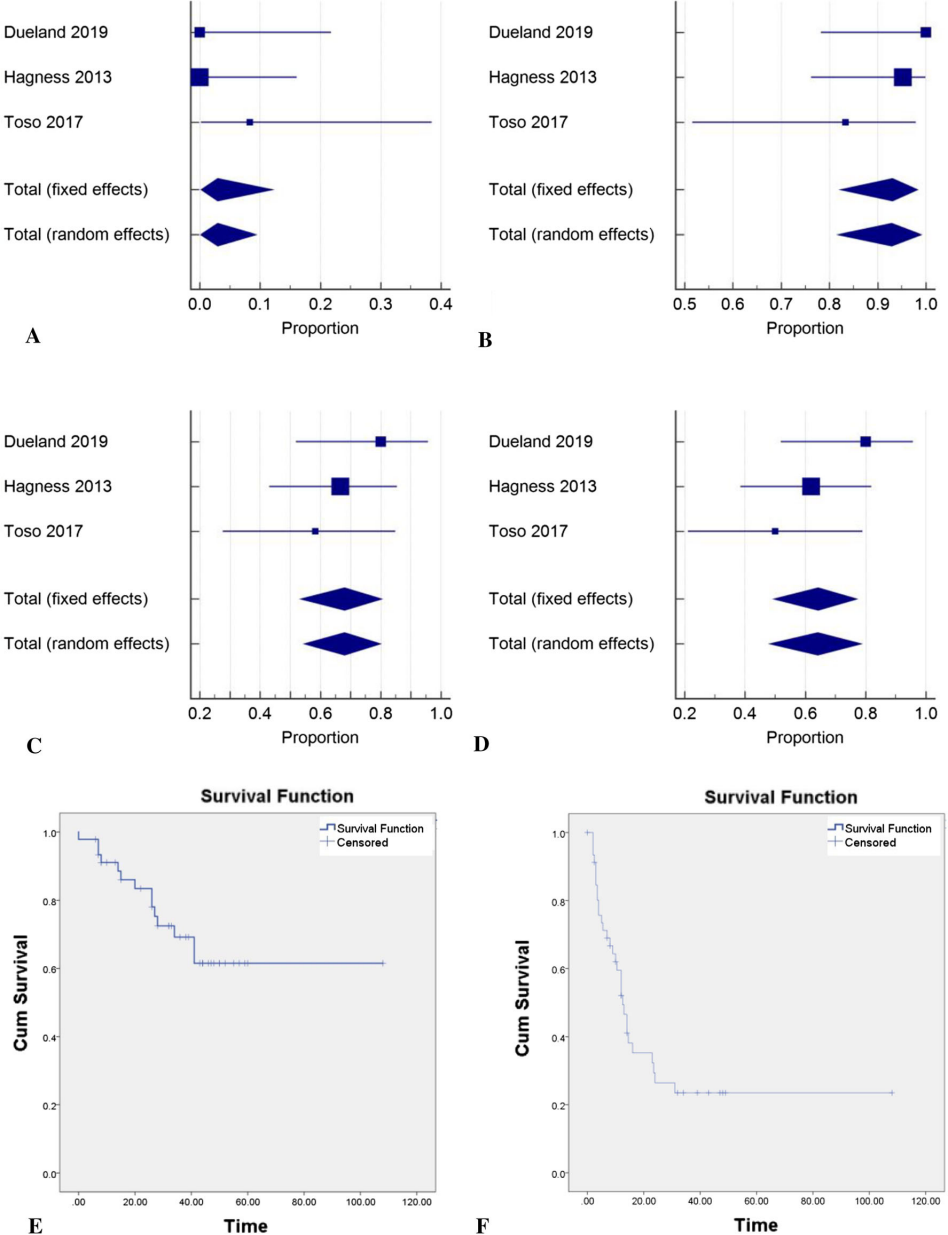
Survival outcomes: **A** 30-day mortality, **B** 1-year survival, **C** 3-year survival, **D** 5-year survival. Kaplan–Meier survival curves: **E** overall survival, **F** disease-free survival

All studies reported complications using the Clavien-Dindo (CD) classification system. Major complication rates (CD grades III-V) were 33.3%, 47.6% and 46.7% (*p* = 0.72, *I*^*2*^ 0.0%). No statistically significant difference was found between 30-day mortality rates (*p* = 0.37, *I*^*2*^ 0.19%) with only one case of 30-day mortality due to uncontrollable haemorrhage (Fig. 3).

### 1, 3 and 5-year survival

One-year survival rates were reported as 83.3%, 95.2% and 100.0% (*p* = 0.22, *I*^2^ 34.5%). Three-year survival rates were reported at 58.3%, 66.7% and 80.0% (*p* = 0.48, *I*^2^ 0.0%). Five-year survival rates were reported at 50.0%, 61.9% and 80.0% (*p* = 0.26, *I*^2^ 26.1%) (Fig. 3). No statistical significance was detected between studies.

### Recurrence, DFS and OS

One-year DFS was reported as 56%, 35% and 53%, respectively, with the proportion free of recurrence at the end of the studies being 41.7% (5/12), 9.5% (2/21) and 53.3% (8/15). The most common site of recurrence in all studies was lung (5, 17 and 5 acses) followed by liver (3, 7 and 1 cases). Treatment for recurrence varied within studies and included a range of chemotherapy, radiotherapy and surgical resection. Five out of six deaths reported by Toso et al. were attributable to disease recurrence. In the SECA-I and SECA-II trials, all reported deaths (*n* = 6, 2) were due to disease recurrence. DFS and OS Kaplan–Meier curves summarising merged data from all studies are demonstrated in Fig. 3.

### Registered trials

Eight trials for LT in CRLM are registered on clinicaltrials.gov at time of writing spanning six countries (Table 2). Three RCTs plus one study with a randomised element [19–22], three single group assignments [23–25] and two non-randomised trials with parallel trials to be used as comparison groups [22, 26]. Interventions include living donor (LD) transplantation [23, 24], deceased donor (DD) transplantation [21, 22, 25, 26], extended criteria donor (ECD) transplantation [20] and liver transplantation with staged/delayed hepatectomy [23, 25]. The RCT comparison arms consist of standard chemotherapy regimens or “best alternate care”.

**Table 2:** Summary of ongoing trials looking at liver transplantation for colorectal liver metastases

Trial identifier	Country	Study Design	Intervention	Estimated enrolment	Start date	Estimated end date	Primary outcome
NCT02597348(22)	France	RCT Multicentre	LT preceded by non-experimental standard chemotherapy	90	2015	2027	5 year overall survival
NCT03488953(26)	Germany	Single group assignment Multicentre	Living donor liver transplant with 2 stage hepatectomy	40	2018	2023	3 year overall survival post second stage hepatectomy
NCT04161092(23)	Sweden	RCT#Multicentre	Extended criteria donor LT + 'best alternate care'	45	2020	2029	5 year overall survival
NCT02864485(27)	Canada	Single group assignment Single centre	Live donor LT + standard chemotherapy regime	20	2016	2023	5 year overall survival, 5 year DFS
NCT01479608(25)	Norway	Non-randomised, parallel assignment (some randomisation open-label) Single centre	1. LT vs liver resection 1:1 randomisation. 2. LT for NRCLM (metachronus). 3. LT for NRCLM (synchronous). 4. LT for NRCLM (synchronous) with expected overall survival 6–12 months	25	2012	2025	10 year overall survival
NCT03494946(24)	Norway	RCT Single centre	LT vs chemoTACE/SIRT or other available options	30	2016	2027	2 year overall survival
NCT03803436(29)	Italy	Non-randomised, parallel assignment Multicentre	Deceased donor LT (comparison group will be COLT-eligible patients who enter TRIPLETE trial—mFOLFOX panitumumab)	22	2019	2024	5 year overall survival
NCT02215889(28)	Norway	Single group assignment Single centre	LT + segment 2/3 resection + delayed hepatectomy	20	2014	2021	Percentage of transplant patients receiving second stage hepatectomy within 4 weeks

RCT (randomised control trial), LT (liver transplantation), NRCLM (non-resectable colorectal liver metastases), TACE (transarterial chemoembolization), SIRT (selective internal radiation therapy), DFS (disease-free survival)

## Discussion

Our analysis of 48 patients is likely to have captured the majority of LT carried out for CRLM during this time period; the ELTR reports 53 liver transplants for CRLM carried out from 2001–2016 (13). All included studies are European and only one of the eight trials registered on clinicaltrials.gov is based outside Europe (Canada, NCT02864485 [24]). Despite heterogeneity both within and between study populations, the results appear concordant with each other.

The 5-year survival for all indications of LT is 71% and has been relatively steady since 2000 [10]. The current five-year survival following LT is 67% for primary liver tumours and 61% for metastatic liver disease [10]. The reported five-year survival rates of 50%, 61.9% and 80% in studies after 1995 show a vast improvement from the 18% seen prior to 1995—at which time, the rate of graft loss in CRLM patients was as high as 44% in the absence of tumour recurrence [8] and a series of 25 patients reported a 30-day mortality approaching 30% [27]. This is consistent with known improvements in LT outcomes due to advancements in surgical technique, better immunosuppressive regimens [28], improvements in the management of advanced colorectal cancer, including chemotherapy regimens [29] and better methods of down-staging tumours [28].

Although the treatment for NRCLM, palliative chemotherapy, has also improved in recent years, the five-year survival remains less than 10% [4, 5]. The same cohort from the SECA-I trial has been directly compared with a corresponding group from the NORDIC VII trial (first line chemotherapy for NRCLM) with five-year survival in the NORDIC VII group of 9%, rising to 19% when only those with the most favourable tumour and disease characteristics were considered [30]. This remains a significantly lower OS when compared with LT studies.

Despite the promising data regarding OS, there remains a high recurrence rate (44–65% in the first year) and DFS reduces rapidly during the first two years following transplant for CRLM. Typically DFS is seen as a good surrogate marker for OS in CRC, however despite the early recurrence seen in this study, OS remains relatively high.

Pulmonary recurrence was the most frequent site of recurrence in all studies, a proportion of which may be the result of undetected micrometastases at time of tumour staging. This is due to the lack of diagnostic methods with adequate sensitivity to detect and characterize very small lesions. Following LT, where lung was the first-site of recurrence the 5-year survival was 72%. 5-year OS from all sites of recurrence was 53% [31].

Interestingly, growth of pulmonary metastases was relatively slow despite immunosuppression and although OS was poorer compared to those without pulmonary metastases, OS remained greater than when compared with outcomes after palliative chemotherapy [32]. We suggest that the link between DFS and OS should be interpreted with caution in the setting of LT for CRLM in upcoming trials, particularly in pulmonary recurrence.

Given the extrahepatic nature of the majority of metastases, control of systemic disease is an important factor. All patients received neo-adjuvant chemotherapy, however of the 48 patients, only four (8.3%) received adjuvant chemotherapy following LT, unfortunately individual outcome data was not reported. Adjuvant chemotherapy following resection of liver metastases has been shown to improve OS [33], however, the side effects of chemotherapy may be exacerbated by immunosuppression [34] and certain chemotherapeutic agents have the potential to increase the risk of rejection [35]. Adjuvant chemotherapy would treat any undetected micrometastases and circulating tumour cells to prevent seeding. Further investigation is required to establish efficacy and safety in this setting, however none of the currently registered trials clearly report the use of adjuvant chemotherapy.

To determine the true impact of disease recurrence and define the relationship between DFS and OS, the publication of long-term survival data is necessary. It is known that the 10-year survival for LT for NET is 46.1% for isolated liver metastases [36] and 12–36% for those who have undergone liver resection for CRLM [37]. It will be interesting to see how this will compare to those who have undergone LT for CRLM.

The apparent improvement in the first five years following LT reported between the SECA-I and SECA-II trials could be explained in part by refined patient selection. The SECA-I trial identified 4 factors: pre-transplant tumour diameter > 5.5 cm (high hepatic tumour load), CEA before LT > 80ug/L, disease progression on chemotherapy and short interval from primary resection to transplant [17]. Although the association of 5-year survival with tumour size was not seen by Toso et al. [16], these factors are consistent with known poor prognostic indicators, including following R0 resection, of CRLM [6, 38].

Scoring systems used to predict recurrence after CRLM resection, such as the Fong Clinical Risk Score (FCRS) [39] or the Oslo Score, proposed by the Norwegian group to identify patients at risk of recurrence after LT, are both based on the above factors and could be used to aid patient selection to obtain survival rates comparable with other indications for LT [40].

The commonality of these factors are all indicative of an aggressive tumour biology–rather than the technical factors which make a tumour unresectable. Patients with a high hepatic tumour load who underwent LT for NRCLM were matched to a group who had resectable disease and who underwent portal vein embolization plus liver resection. OS was significantly higher in the LT group which may indicate that technically resectable patients may also benefit from transplantation, an area which needs further research [41].

Given the scarcity of resources, defining the patient population who will most benefit from liver transplantation is a key step and refinement of prognostic indicators in coming trials could aid decision-making when national graft allocation is considered. It has been argued that with strict criteria, only a very small subset of patients with NRCLM will be eligible for transplantation; the SECA-II trial recruited 15 patients over five years from a catchment area with a population of five million [18] which would expand if resectable patients were also considered.

To address this, trials are looking at living donation (NCT03488953 [23], NCT02864485 [24]) and extended criteria donors (NCT04161092 [20]) as a way to expand the donor pool. Currently living donors account for just 2–3% of donors for primary and secondary liver malignancies [36]. Reported outcomes for ECD (an arm of the SECA II trial) had a shorter DFS and worse OS compared to those who underwent standard graft transplantation, however they had more advanced disease. 1/10 was re-transplanted for graft failure [42]. A small case series in South Africa has used ECD with patients who meet the SECA I inclusion criteria with an 80% (4/5) survival (death due to recurrent disease) with a median follow up of 38 months and a 100% (5/5) recurrence rate with a median recurrence of six months [43]. No trials are currently looking into those with technically resectable disease.

Much of the literature published in recent years has come out of Oslo, Norway, where the deceased donor pool is relatively large with a short transplant waiting list. If the currently registered trials hit their enrollment targets, the data for nearly 300 further patients will become available over the next 10 years, however this may still leave some matters unaddressed.

## Conclusion

Although current evidence suggests a survival benefit conferred by LT in NRCLM, the ethical implications of organ availability and allocation demand rigorous justification. The current evidence is encouraging but refers to a small patient population. Larger randomized studies with more longitudinal data are needed and the refinement of patient selection is critical to improve DFS and OS. Concomitant improvements in the management of patients following liver resection and of palliative chemotherapy regimens is paramount.
